# Comparison of different concentrations of ropivacaine in epidural anesthesia for percutaneous transforaminal endoscopic discectomy: a randomized controlled trial

**DOI:** 10.1186/s12871-024-02588-5

**Published:** 2024-07-04

**Authors:** Fengyan Shen, Yuju Pu, Zhiming Lan, Lijun Fu, Yan Zhang, Shenghua He, Zengping Huang

**Affiliations:** 1 Department of Anesthesiology, Shenzhen Traditional Chinese Medicine Hospital, Guangzhou University of Traditional Chinese Medicine, Shenzhen, 518033 Guangdong China; 2 Department of Orthopedics, Shenzhen Traditional Chinese Medicine Hospital, Guangzhou University of Traditional Chinese Medicine, Shenzhen, 518033 Guangdong China

**Keywords:** Percutaneous transforaminal endoscopic discectomy, Epidural anesthesia, Ropivacaine, Analgesia, Randomized controlled trial

## Abstract

**Background:**

This study investigated the optimal concentration of ropivacaine epidural anesthesia for clinical use in percutaneous transforaminal endoscopic discectomy (PTED) by comparing the effects of different concentrations.

**Methods:**

Seventy patients scheduled for their first PTED procedure were enrolled in this randomized controlled trial. Patients were randomized to receive ropivacaine at varying concentrations (0.3% or 0.4%). Primary outcome measures included the numeric rating scale (NRS) and hip extension level (HEL). Secondary outcome measures included intraoperative fentanyl dosage and postoperative complications.

**Results:**

One patient withdrew due to severe postoperative complications. The remaining 69 patients were allocated to the 0.3% (*n* = 34) and 0.4% (*n* = 35) groups, respectively. Baseline characteristics showed no significant differences between the two groups (*P* > 0.05). The NRS score was significantly lower in the 0.4% group than in the 0.3% group (*P* < 0.01), whereas the HEL score was significantly higher (*P* < 0.001). The average fentanyl dose in the 0.4% group was significantly lower than that in the 0.3% group (*P* < 0.01). Postoperative complications occurred in five and two patients in the 0.3% and 0.4% groups, respectively.

**Conclusion:**

Although 0.4% ropivacaine (20 mL) impacts muscle strength, it does not impede PTED surgery. Given its effective analgesic properties and few postoperative complications, 0.4% ropivacaine can be considered a preferred dose for PTED.

**Trial registration:**

This study was registered with the Chinese Clinical Trials Registry (Registration number: ChiCTR2200060364; Registration Date: 29/5/2022) and on chictr.org.cn (https://www.chictr.org.cn/showproj.html?proj=171002).

## Background

Epidural anesthesia (EA) involves injecting a local anesthetic into the epidural space, which blocks the spinal nerve root and induces temporary paralysis in the corresponding controlled area [1]. Theoretically, EA can be used for surgery on any body part except the head. In recent years, a continuous expansion of EA application in emerging technological surgeries, such as percutaneous transforaminal endoscopic discectomy (PTED), has been observed [2].

PTED removes lesion tissue and hyperosteogeny from the nucleus pulposus, nerve root, or dural sac. Spine surgeons are increasingly favoring this technique due to its minimal complications, high safety, and rapid postoperative recovery [3, 4]. However, PTED’s proximity to the nerve root within the operation channel puts it at risk of accidental damage.

Therefore, the current anesthesia method frequently employed for PTED involves lumbar local anesthesia combined with intensive intravenous anesthesia. This approach aims to prevent nerve root injury by allowing for real-time feedback. This feedback includes monitoring for sensory changes, such as numbness or distension, and observing lower limb movement. However, local anesthesia combined with intensive intravenous anesthesia is often insufficient, leaving patients less tolerant of the intense pain or nerve root stimulation caused by surgery [5].

Ropivacaine, a long-acting amide local anesthetic, reversibly blocks nerve impulse conduction through sodium channels, similar to other local anesthetics. This mechanism provides both anesthetic and analgesic effects. Higher concentrations induce surgical anesthesia, whereas low concentrations induce a sensory block (analgesia) coupled with limited non-progressive motor nerve block [6, 7]. Given these properties, ropivacaine is widely applied in labor analgesia and is suited to the specific anesthetic needs of PTED. Consequently, anesthesiologists have begun using ropivacaine EA for PTED.

Recent meta-analysis findings highlight the superior analgesic advantages of EA in PTED compared with local anesthesia, along with a reduced incidence of postoperative complications [8, 9]. These findings suggest that using EA in PTED has potential advantages. Currently, there is no standardized approach for using ropivacaine EA in PTED, and the complex relationship between drug concentration and compatibility methods adds to the challenge. Therefore, this study aimed to determine the optimal concentration for practical use by comparing the effects of different ropivacaine EA concentrations on PTED. This study will provide valuable data for establishing a unified standard for using ropivacaine EA in PTED procedures.

## Methods

### Ethics statement

This study complied with the principles of medical ethics stated in the Declaration of Helsinki and has been approved by the Ethics Committee of Shenzhen Traditional Chinese Medicine Hospital (Approval number: K2022-015-02). All participants were informed in detail of the trial and signed consent forms.

### Study participants

This study included patients admitted to the Department of Orthopedics at Shenzhen Traditional Chinese Medicine Hospital between May 2022 and December 2022, who were scheduled for their initial PTED procedure and voluntarily consented to receive EA. The patients’ age ranged from 18 to 65 years, with a physical status classification of I and II according to the American Society of Anesthesiologists.

A double-blind, prospective, randomized controlled trial was conducted, allocating patients to receive ropivacaine at either 0.3% or 0.4% concentration.

Patients were excluded if they had prior lumbar surgery, trauma, or deformity, neurological defects due to non-lumbar disc herniation, abnormal coagulation function, communication barriers, or were pregnant or menstruating. The data exclusion criteria included the use of unauthorized medications, incomplete data hindering efficacy and safety assessment, surgery exceeding 2 h, and unforeseen conditions or severe postoperative complications.

This study retrospectively analyzed data from 669 PTED procedures performed at Shenzhen Traditional Chinese Medicine Hospital between January 2018 and November 2021 using ropivacaine EA. Among these, 71 patients received 0.3% ropivacaine, 168 patients received 0.4% ropivacaine, and the remaining patients received varying concentrations (0.325%, 0.35%, or 0.375%) of ropivacaine. The probability of requiring intraoperative remedial analgesia with 0.3% ropivacaine was 64.8%, compared to 23.2% with 0.4% ropivacaine.

Sample size calculation, aiming for 90% power and a 5% significance level, was based on the aforementioned remedial analgesia rates using SigmaStat software (version 3.5, Jandel Scientific Software, USA). This analysis determined a minimum of 33 patients per group. To ensure adequate safety margins, the study enrolled 35 patients in each group. All participants provided informed consent before the trial commenced.

### Trial procedure

In the 24 h preceding surgery, Investigator A conducted preoperative assessments, evaluated potential participants, and enrolled those who met the criteria. They then recorded basic patient information and submitted it to Anesthesiologist A. Anesthesiologist A used a dynamic randomization system based on *R* software (version 4.2.1; The *R* Project for Statistical Computing) to allocate patients to groups, considering factors such as sex, age, and number of lumbar segments.

Anesthesiologist A prepared the ropivacaine (JIABO PHARMACEUTICAL, Guangdong, China) according to the randomization group (20 mL) but without labeling the concentration. The syringe containing the medication was then transferred to Anesthesiologist B.

Anesthesiologist B began the procedure by injecting 5 mL of the medication at the designated puncture site (the second intervertebral space above the surgical lumbar segment). The catheter was inserted in an upward direction to a depth of 3–5 cm. Following epidural catheterization, the patient was positioned supine, and vital signs (respiration, heart rate, and blood pressure) were monitored for 5 min. After confirming no abnormalities, the remaining ropivacaine was injected into the epidural space in two doses, with a 5-min interval between administrations. After observing the patient for 15 min without complications and confirming consistent anesthesia levels, the patient was repositioned, and prepared for surgery with disinfection procedures. All patients underwent surgery performed by two prescribing orthopedic doctors.

If a patient experienced significant pain, nerve root irritation (numbness or tingling), or required pain relief during surgery, Anesthesiologist B promptly administered fentanyl (0.05 mg, HUMANWELL HEALTHCARE, WUHAN, China) intravenously, along with dextrometomidine (initial dose: 0.5 µg/kg over 10 min, followed by a maintenance dose: 0.5 µg/kg/h; JIANGSU HENGRUI MEDICINE, Lianyungang, China) via intravenous infusion. If the patient could not tolerate this medication, additional fentanyl (0.05 mg each time) was administered until pain was adequately controlled. Intraoperative data were recorded by Investigator B.

Four hours after surgery, routine administration of analgesics (diclofenac sodium sustained-release tablets, 75 mg bid, oral; JIANGXI RENHE PHARMACEUTICAL, Zhangshu, China) was initiated. Investigator A conducted postoperative follow-ups at 24 h and recorded relevant data. Investigators A and B submitted their records to Anesthesiologist A for entry into the secure database.

Anesthesiologist A was the only person aware of the group allocations. All other investigators maintained their assigned roles, refrained from discussing patient information that could reveal group allocation, and did not interfere with each other’s work until the unblinding process. Once data collection was complete, Anesthesiologist A submitted a de-identified research form (without basic patient information) to Investigators A and B for statistical analysis. The protocol included emergency unblinding procedures, where intraoperative rescue or treatment fell outside the study’s scope. This allowed the on-site doctors to clarify participant grouping and administer necessary treatment to patients.

### Outcome measures

The primary outcome measures were the numeric rating scale (NRS) for pain and the hip extension level (HEL) for muscle strength. The NRS uses a 0–10 scale (with 11 points) to represent pain or nerve root irritation intensity. Zero indicates no pain or irritation, and 10 represents the worst pain imaginable. Patients self-assessed their pain or irritation levels during and 24 h after surgery. The intraoperative NRS was the highest score recorded during surgery, while the postoperative NRS was measured at the 24 h time point.

Owing to the compression of the lumbar intervertebral disc, the muscle strength of the affected side may diminish. Therefore, HEL assesses muscle strength on the contralateral side. It has six levels, ranging from no muscle contraction (level 0), muscle contraction (level I), inability to counteract gravity, except when in a lateral lying position, where hip joint extension can be achieved with the help of the examiner (level II), assuming the prone position and accomplishing hip joint extension while countering gravity, yet not against external forces (level III), capacity to perform hip joint extension under specific external forces (level IV), and normal muscle strength, enabling full resistance against external force to achieve hip joint extension (level V). HEL measurements were taken before surgery, immediately after surgery, and 24 h post-surgery.

The secondary outcome measures included intraoperative fentanyl dosage and postoperative complications.

### Statistical analyses

Statistical analyses were performed using Statistical Package for the Social Sciences 23 (International Business, Armonk, NY). Descriptive data were presented as mean ± standard deviation, median, or interquartile range. The t- or rank-sum test was applied to compare two sets of independent data. Repeated-measures analysis of variance was employed for parameters measured repeatedly. Chi-square tests were used to analyze categorical data. *P* < 0.05 indicates a statistically significant difference.

## Results

### Baseline characteristics

**Fig. 1:**
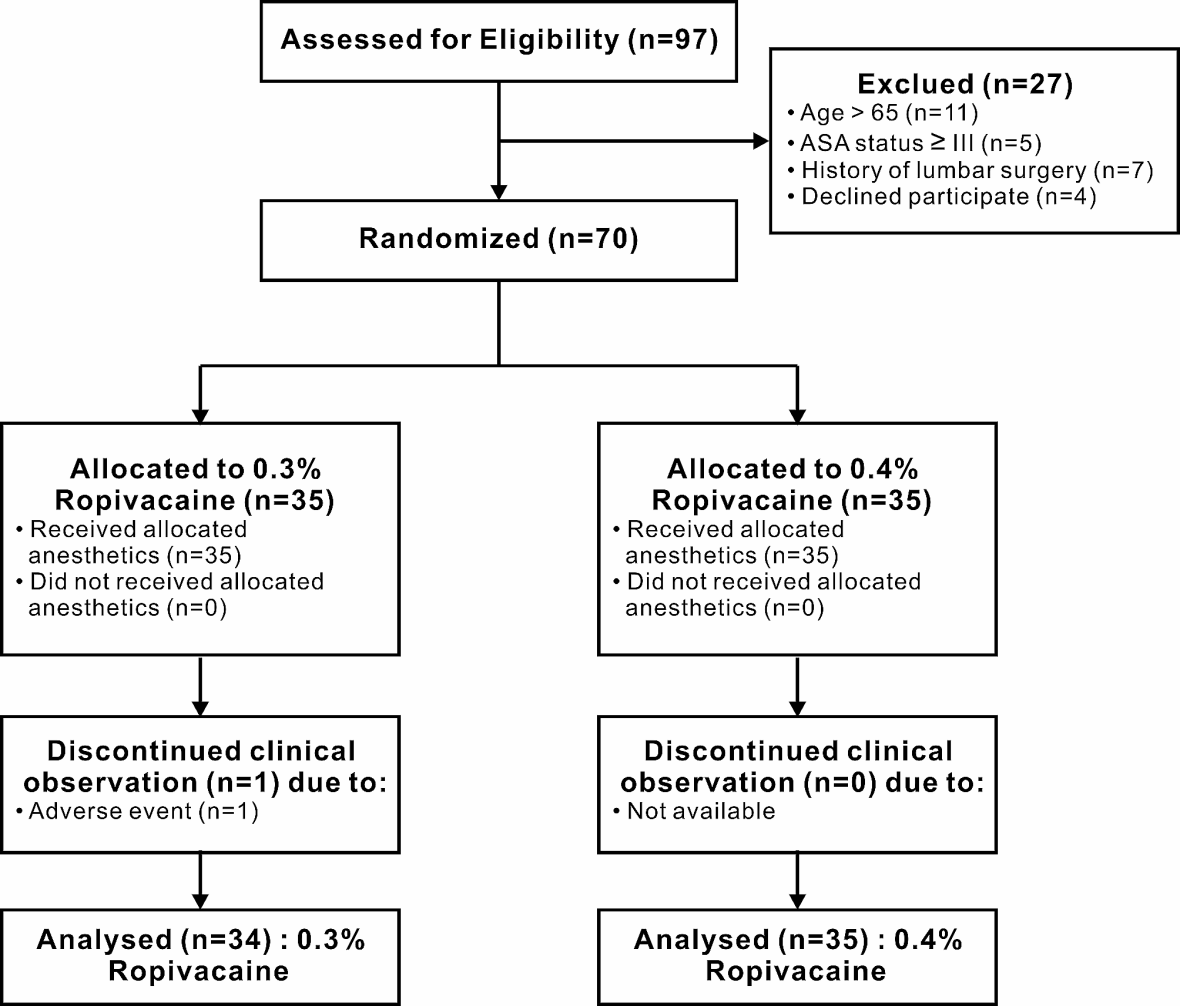
Flow diagram of the trial. ASA, American Society of Anesthesiologists

Seventy patients were allocated to either the 0.3% or 0.4% ropivacaine concentration group based on their randomization. One patient experienced severe postoperative complications suspected to be spinal cord hypertension, requiring emergency symptomatic treatment. Consequently, this patient’s data were excluded from the final analysis according to the predefined criteria. Therefore, a total of 69 patients were included in the final analysis (Fig. 1). Baseline characteristics between the two groups showed no statistically significant differences (Table 1).

**Table 1:** Baseline characteristics

Index	Group		
	0.3%	0.4%	*P*-value
**n**	34	35	—
**Sex (Male/Female)**	21/13	22/13	0.925
**Age**	44.1 ± 11.6	44.6 ± 10.0	0.853
**Body mass index**	24.3 ± 3.2	24.0 ± 2.8	0.734
**Number of lumbar segments** **(1 segment/2 segments)**	31/3	33/2	0.618
**Preoperative heart rate**	73.1 ± 10.3	76.4 ± 10.4	0.202
**Preoperative mean arterial pressure**	95.0 ± 11.3	100.3 ± 18.2	0.150
**Operation duration**	56.8 ± 17.1	62.6 ± 21.0	0.213

Pearson’s chi-squared test was used for gender and number of lumbar segment comparisons; Student’s t-test was used for age, body mass index, heart rate, mean arterial pressure, and operation duration comparisons

### Primary outcome measures

In the 0.3% group, six patients reported minimal pain (NRS = 0), whereas this number increased to 12 patients in the 0.4% group. The 0.4% group also had a significantly lower mean NRS score. The NRS scores in both groups significantly decreased 24 h after surgery, with no significant difference observed between the groups (Table 2).

**Table 2:** Comparison of NRS

Process	Group	Mean (IQR)	*P*-value		
			Mann-whitney rank sum test	Wilcoxon signed rank test	
**Intraoperative NRS**	0.3%	3 (2, 6)	0.004	0.000	*VS* Postoperative NRS
	0.4%	2 (0, 3)		0.001	
**Postoperative NRS**	0.3%	0 (0, 2)	0.559	—	
	0.4%	0 (0, 2)			

Mann-Whitney Rank Sum Test was used to compare the NRS in different groups at the same process; Wilcoxon Signed Rank Test was used to compare the NRS in the same group at different processes. Intraoperative NRS was the worst value of NRS during the surgery, while the postoperative NRS was the one on the time point of 24 h after surgery. NRS, Numerical Rating Scale; IQR, interquartile range

Thirteen patients in the 0.3% group maintained unaffected HEL (level V), while all patients in the 0.4% group experienced a decrease in HEL. The mean HEL in the 0.4% group was significantly lower than the 0.3% group, as confirmed by statistical analysis. However, HEL in both groups recovered to near-normal levels 24 h after surgery (Table 3).

**Table 3:** Strength of the contralateral hip extension level (HEL)

Process	Group	Mean (IQR)	*P*-value		
			Mann-whitney rank sum test	Wilcoxon signed rank test	
**HEL before surgery**	0.3%	5 (5, 5)	1.000	0.000	*VS* HEL immediately after surgery
	0.4%	5 (5, 5)		0.000	
**HEL immediately after surgery**	0.3%	4 (4, 5)	0.000	0.000	*VS* HEL at 24 h after surgery
	0.4%	3 (2, 4)		0.000	
**HEL at 24 h after surgery**	0.3%	5 (5, 5)	0.324	1.000	*VS* HEL before surgery
	0.4%	5 (5, 5)		0.317	

Mann-Whitney Rank Sum Test was used to compare the HEL in different groups at the same process; the Wilcoxon Signed Rank Test was used to compare the HEL in the same group before, immediately after, and 24 h after surgery. IQR, interquartile range

### Secondary outcome measures

**Fig. 2:**
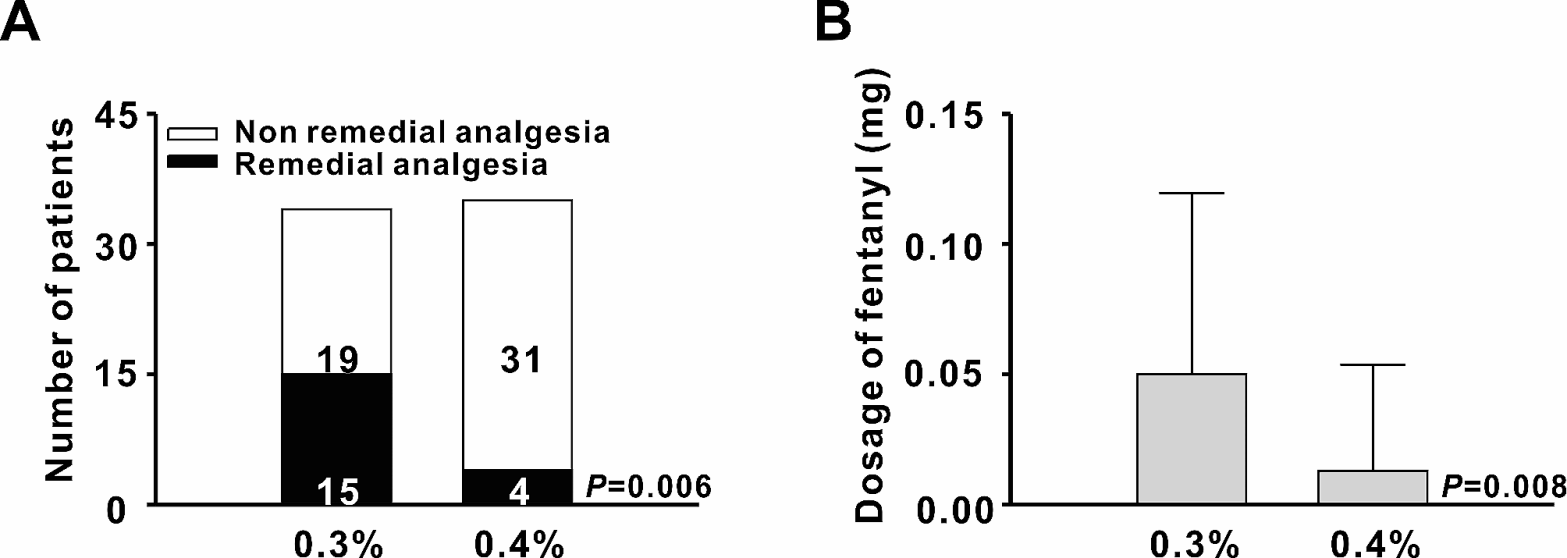
Comparison of remedial analgesia. **(a)** Ratio of remedial analgesia. Pearson’s chi-squared test. **(b)** Amount of fentanyl used during surgery. Student’s t-test

During surgery, fifteen patients in the 0.3% group required fentanyl, compared to only four patients in the 0.4% group (Fig. 2a). The average fentanyl dosage in the 0.3% and 0.4% groups was 0.05 ± 0.07 mg and 0.01 ± 0.04 mg, respectively, with a significant difference between the groups (Fig. 2b).

Regarding postoperative complications, the 0.3% group had two patients experiencing voiding dysfunction, two patients with nausea and vomiting, and one patient with abdominal distension. The 0.4% group had one case of skin allergy and one patient reported persistent mild lower limb numbness.

## Discussion

Our institution conducted a retrospective study comparing the safety and effectiveness of PTED under EA and general anesthesia [10]. They found no significant difference in complication rates between the two anesthesia methods. However, we recommend that junior surgeons prioritize using EA for PTED procedures. This allows them to receive real-time patient feedback, potentially reducing the risk of nerve injury and minimizing radiation exposure during surgery.

Traditionally, many PTED procedures are performed under local anesthesia combined with neuroleptic medication (produced by opioids and/or neuroleptics) [8, 9]. This study excluded neuroleptics to promote better patient cooperation during surgery. While opioid analgesics are essential for managing severe pain, their use carries risks for both patients and society, including misuse, abuse, inattention, addiction, suicide, and overdose fatalities [11]. Additionally, high opioid doses can cause serious adverse effects on various bodily systems, including the central nervous system, respiratory system, cardiovascular system, endocrine system, digestive system, and urinary system [12].

Compared to local anesthetics with opioids, EA offers potentially superior pain relief during PTED while minimizing postoperative complications, likely due to reduced opioid use [8, 9]. The incidences of postoperative complications in this study are 14.7% and 5.7%, respectively. While the 0.4% ropivacaine EA group showed a lower complication rate, the overall number of complications in both groups was too small to reveal statistical differences.

Several recent studies have investigated the concentration of ropivacaine used for EA in PTED, reporting a range of 0.12–0.4% [5, 8, 9, 13–17]. However, in our pilot study, 0.2% of ropivacaine administered via EA did not provide adequate pain relief during PTED. Patients continued to experience pain after routine EA, necessitating additional interventions such as switching to general anesthesia or applying local anesthetics at the surgical site.

A two-stage, biased-coin design study by Hu et al. identified 0.294% as the minimum effective concentration for ropivacaine for EA during PTED. Another study by the same group demonstrated that 0.4% ropivacaine EA is superior to local or intravenous anesthesia for PTED, offering a low incidence of non-tactile nerve block, optimal pain relief, and a stable intraoperative circulatory state [18, 19]. It is important to note that higher ropivacaine concentrations can decrease muscle strength, potentially hindering the surgeon’s ability to assess nerve injuries during PTED. Therefore, this study compared the concentrations of 0.3% and 0.4%.

To determine the sample size, this study considered our institution’s prior experience using ropivacaine EA in PTED. The probabilities of requiring additional intraoperative remedial analgesia were significantly higher with 0.3% ropivacaine EA (64.8%) compared to 0.4% (23.2%). Conversely, the probabilities of not requiring additional medication were 35.2% and 76.8% for the 0.3% and 0.4% groups, respectively.

These differences may be due to variations in surgeon and anesthesiologist experience and skill level. Additionally, patients in this study were well-informed about the procedure, allowing them to better distinguish between nerve root irritation symptoms and pain. This likely reduced the unnecessary use of supplementary analgesics.

Our primary outcome measures indicate that 0.4% ropivacaine EA provides superior pain relief compared to 0.3% ropivacaine. This is further supported by the significant reduction in opioid use observed in the 0.4% group (11.4%) compared to the 0.3% group (42.9%). While this study examined patient response to pain through heart rate and mean arterial pressure measurements, no significant differences were observed between the groups. This might be due to external factors like emotional state, personal health, and drug reactions, which can influence heart rate and blood pressure changes [20, 21].

Although rare in studies on ropivacaine EA and PTED, this study investigated perioperative muscle function. While the HEL accurately reflected changes in core lower limb muscle strength before and after surgery, surgeon instructions primarily focused on the distal lower limb, where muscle strength remained inadequate. Therefore, future studies should consider including assessments of both core and distal muscle strength, along with surgeon-reported patient cooperation levels.

These results strongly suggest that a 0.4% ropivacaine solution is more effective than 0.3% for EA in patients undergoing PTED.

Patient instructions during PTED can help minimize nerve root injuries, but minor injuries may still occur. These can sometimes become evident under intraoperative lavage fluid pressure and potentially induce symptoms of spinal cord hypertension [22]. This study identified two patients with suspected spinal cord injuries. One patient experienced persistent mild lower limb numbness. The other patient presented with severe numbness in both lower limbs, whole-body muscle tremors, and continuous elevation in heart rate and blood pressure, along with irritability. After repeated sedation and dehydration, the condition of the latter patient gradually improved. Due to this unplanned development requiring additional treatment, the patient’s data was excluded from the analysis. Therefore, preventing spinal cord hypertension remains a crucial concern in PTED.

## Conclusion

Ropivacaine EA shows promise for PTED, although a 0.4% concentration (20 mL) may slightly affect muscle strength. However, this effect does not hinder PTED surgery. Therefore, due to its strong analgesic effect and minimal postoperative complications, 0.4% ropivacaine is recommended as the standard dose for PTED.

## Data Availability

The datasets used and analyzed during the current study are available from the corresponding author upon reasonable request.

## Abbreviations

EA: Epidural anesthesia
HEL: Hip extension level
NRS: Numeric rating scale
PTED: Percutaneous transforaminal endoscopic discectomy.
